# Multi-view state-space modeling for zero-shot circRNA–miRNA interaction prediction

**DOI:** 10.1093/bioinformatics/btag797

**Published:** 2026-10-26

**Authors:** Meng-Meng Wei, Lei Wang, Yu-An Huang, Zhi-An Huang, Peng-Wei Hu, Zhu-Hong You

**Affiliations:** School of Computer Science and Technology/School of Artificial Intelligence, China University of Mining and Technology, Xuzhou, 221116, China; School of Computer Science and Technology/School of Artificial Intelligence, China University of Mining and Technology, Xuzhou, 221116, China; School of Computer Science, Northwestern Polytechnical University, Xi’an, 710129, China; Research Office, City University of Hong Kong (Dongguan), Dongguan, 523808, China; Xinjiang Technical Institute of Physics and Chemistry, University of the Chinese Academy of Sciences, Urumqi, 830011, China; School of Computer Science, Northwestern Polytechnical University, Xi’an, 710129, China

## Abstract

**Motivation:**

CircRNA–miRNA interactions (CMIs) play regulatory roles in human diseases and provide clues for therapeutic target discovery. However, most existing prediction methods rely on circRNA–miRNA pairs and overlook disease-level regulatory contexts, which limits their ability to characterize cross-pathway dependencies in zero-shot inference.

**Results:**

We propose MV-S^2^M, a multi-view state-space modeling framework that integrates molecular attributes to enhance relational reasoning, enabling zero-shot CMI prediction. Specifically, MV-S^2^M performs structure-aware diffusion to enrich molecular priors and constructs disease-mediated semantic subgraphs. It then incorporates state-space modeling to capture long-range dependencies and view-specific regulatory patterns. The learned multi-view representations are fused through a linear projection and fed into an MLP for CMI prediction. Extensive experiments on multiple benchmark datasets demonstrate that MV-S^2^M achieves state-of-the-art performance under both zero-shot and conventional settings. Cold-start evaluations reveal distinct generalization behavior across different unseen-entity settings. Case studies illustrate the contribution of meta-path-guided semantic subgraphs to CMI prediction.

**Availability and implementation:**

The code and data are publicly available at https://github.com/591286260/MV and https://doi.org/10.5281/zenodo.22858403.

## 1 Introduction

Circular RNAs (circRNAs) and microRNAs (miRNAs) are two major classes of non-coding RNAs (Awata *et al.* 2025). Extensive studies have shown that circRNAs function as miRNA sponges and interact with RNA-binding proteins (Cao *et al.* 2024). These activities have been implicated in diverse pathological processes, including tumorigenesis, metabolic disorders, and neurological diseases (Tan *et al.* 2026). Therefore, accurate prediction of circRNA-miRNA interactions (CMIs) is critical for post-transcriptional regulatory analysis and downstream biomedical applications. However, experimentally validated CMIs are still scarce, making it challenging to identify unobserved interactions from existing biological knowledge.

Recent computational efforts have explored a wide range of strategies for CMI prediction. Early approaches relied on matrix factorization to exploit latent association patterns within interaction networks (Fang and Lei 2019, Wang *et al.* 2023a). Subsequent deep learning approaches have leveraged graph neural networks to learn representations from biological networks, enabling the modeling of complex dependencies among network entities (Wang *et al.* 2024a, Cao *et al.* 2025). Although these developments have substantially improved predictive performance, their learned representations are constrained by the coverage of known CMIs, limiting their ability to generalize beyond observed interactions.

To alleviate the dependence on known CMI links, auxiliary biological relations have been incorporated to provide evidence for relational inference. However, effectively integrating such auxiliary information remains challenging (Zhou *et al.* 2025). Recent biomedical representation learning methods have increasingly integrated complementary molecular, spatial, and structural information to obtain more informative representations (Lan *et al.* 2026). In existing heterogeneous networks, node representations are often initialized from independent molecular attributes (Ma *et al.* 2023). Consequently, these isolated priors may fail to reflect the structural dependencies among circRNAs, diseases, and miRNAs, leading to insufficient exploitation of topology-aware contextual information (Hashemi *et al.* 2025).

Beyond integrating node attributes with relational structures, reasoning over heterogeneous relations introduces additional challenges (Zhu *et al.* 2026). Different relational paths encode diverse semantic patterns, and directly aggregating these relations may obscure their specific structural meanings (Niu *et al.* 2025b, Zhang *et al.* 2025). Moreover, dependencies among entities may extend beyond local neighborhoods, while conventional neighborhood-based aggregation schemes are mainly designed for short-range message passing and may struggle to capture long-range relational dependencies (Chen *et al.* 2025). Although attention-based graph models provide a mechanism for modeling global interactions, their explicit computation of pairwise interactions often incurs substantial computational costs when applied to large relational structures.

To address these challenges, we propose MV-S^2^M, a multi-view state-space modeling framework for zero-shot CMI prediction. MV-S^2^M enables zero-shot inference of unobserved CMIs by exploiting disease-mediated relational information and molecular priors. First, a structure-aware diffusion process propagates molecular priors over the heterogeneous network, integrating intrinsic molecular properties with topological information. Then, disease-mediated semantic subgraphs are constructed and modeled with state-space modeling to capture long-range dependencies while preserving view-specific regulatory patterns. Finally, the learned representations are fused through an MLP for CMI prediction. The contributions of this study are summarized as follows:

- We introduce a sparse relation-level CMI inference setting and construct the CMI-Zero benchmark, where target CMIs are excluded from graph representation learning to evaluate generalization using auxiliary biological information.
- We propose MV-S^2^M, a multi-view state-space modeling framework that couples structure-aware diffusion with disease-mediated semantic modeling to enable zero-shot CMI prediction beyond observed interactions.
- Extensive experiments on three benchmark datasets demonstrate that MV-S^2^M consistently outperforms competitive baselines for CMI prediction. Ablation and case studies validate the effectiveness of the proposed framework and reveal the contribution of disease-mediated semantic information.

## 2 Related work

### 2.1 Zero-shot learning

A growing body of work has highlighted the potential of zero-shot learning for biomedical applications (Wang *et al.* 2023b). Early efforts explored zero-shot inference through semantic representation transfer across related entities. For example, Xu *et al.* (2023) introduced BioTranslator, which maps diverse biological modalities into a unified textual semantic space to support zero-shot prediction of unseen cell types, protein functions, and drug targets. In molecular association prediction, Wang *et al.* (2024b) proposed ZeroDDI to recognize unseen interaction types via semantic representation alignment in a shared latent space. ProBID-Net leverages structural information from protein interfaces to perform zero-shot prediction of mutation-induced changes in protein-protein binding affinity (Chen *et al.* 2024). ProtNote combines protein sequences with free-form functional descriptions to enable zero-shot prediction of previously unseen Gene Ontology annotations (Char *et al.* 2025). However, existing zero-shot approaches mainly rely on semantic alignment or auxiliary features, while how to exploit complex relational contexts for unseen biological interaction inference remains underexplored.

### 2.2 State-space modeling

Recent developments have demonstrated the broad applicability of state-space modeling across diverse settings that involve sequential structure and relational dependencies. Ren *et al.* (2025) proposed Samba, a hybrid architecture that integrates selective state-space modeling with sliding-window attention to learn global context and local dependencies in long sequences. Li *et al.* (2024) proposed GraphSSM, which incorporates graph Laplacian regularization into a continuous-time state-space formulation to jointly model graph topology and temporal dynamics. More recently, Cheng *et al.* (2025) developed SourceDetMamba, a graph-aware state-space model for sequential hypergraphs that augments state updates with topological signals to model propagation behavior and higher-order interactions. Collectively, these studies suggest that state-space modeling provides a viable foundation for capturing complex dependencies in sequential and structured data.

### 2.3 CMI prediction

Several CMI prediction methods utilize multi-source biological information to improve entity representations. BioKG-CMI constructs a biological knowledge graph by integrating sequence semantics, spatial proximity, and logical rules learned via knowledge graph embedding, enhancing circRNA and miRNA representations (Wei *et al.* 2024). Beyond pairwise modeling, Yin *et al.* introduced a multi-relational hypergraph framework that models higher-order correlations and contextual interactions, enabling richer structural representations (Yin *et al.* 2026). GraCMI employs matrix factorization-based multi-hop structural modeling over cancer-related networks to capture both intrinsic molecular properties and global topological dependencies (Wei *et al.* 2026). DGCLCMI integrates collaborative filtering signals with sequence encoders through a graph collaboration learning mechanism, resulting in more discriminative representations for CMI prediction (Cao *et al.* 2025).

## 3 Methodology

As illustrated in Fig. 1, MV-S^2^M integrates molecular priors into disease-mediated semantic subgraphs and models their dependencies to support zero-shot CMI prediction. Specifically, circRNA functional similarities, disease ontology semantics, and miRNA sequence embeddings are first propagated over a heterogeneous network via structure-aware diffusion. The diffusion-enhanced representations are then organized into complementary meta-path-guided semantic views and encoded via state-space modeling to capture long-range dependencies. Finally, multi-view representations are fused and fed into an MLP for CMI prediction.

**Figure 1:**
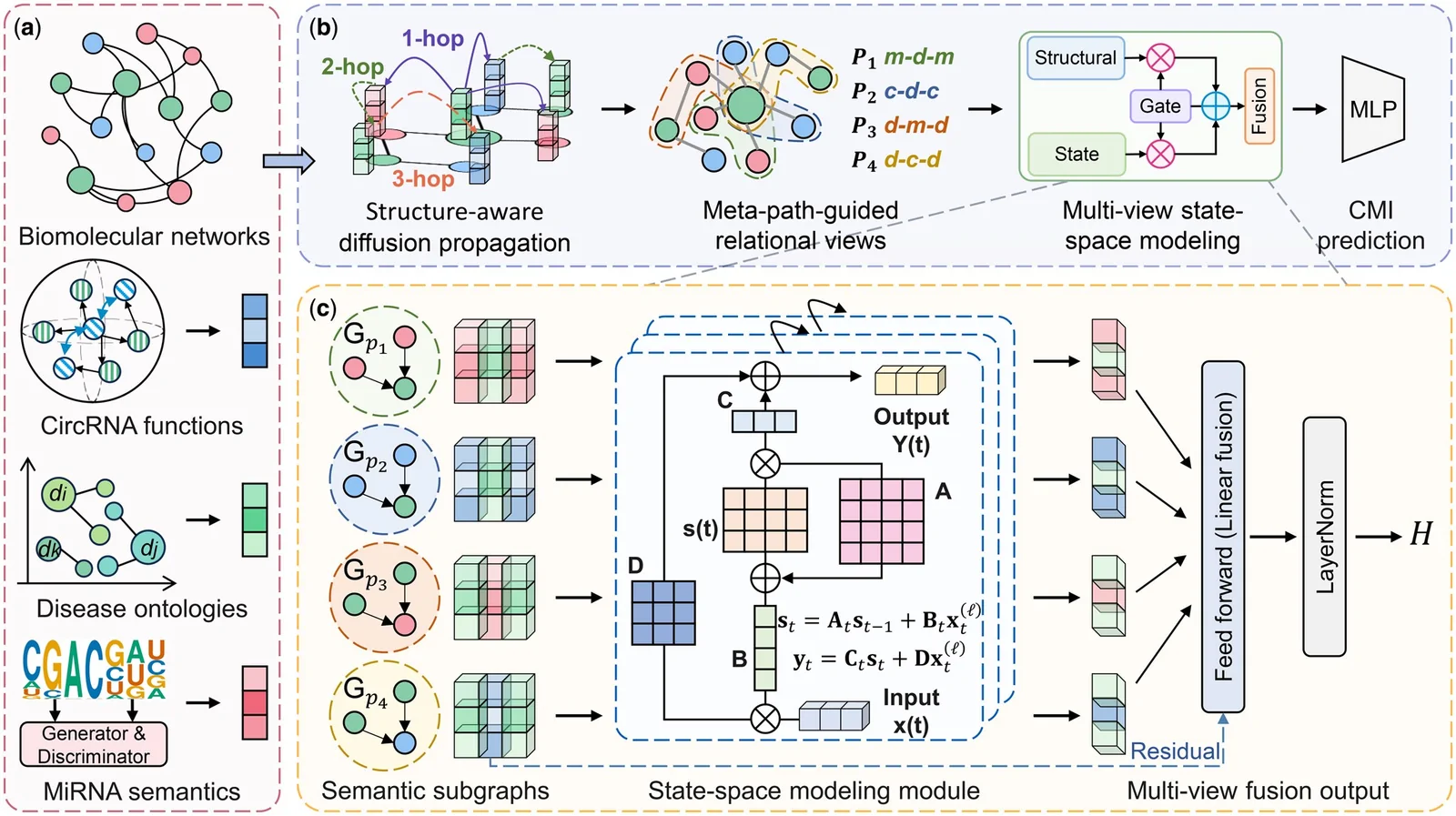
Overall framework of MV-S^2^M. (a) Construction of molecular priors from circRNA, disease, and miRNA data. (b) Diffusion-enhanced molecular priors are used to construct meta-path-guided semantic subgraphs, which are modeled via multi-view state-space modeling, followed by an MLP prediction. (c) Each subgraph is encoded by state-space layers, and the resulting representations are fused through a linear feed-forward layer.

### 3.1 Molecular prior construction

MV-S^2^M constructs molecular priors for circRNAs, diseases, and miRNAs from functional, ontological, and sequence information, respectively.

#### 3.1.1 CircRNA functional similarity calculation

To capture functionally coherent patterns among circRNAs, we construct a Gaussian interaction profile kernel (GIPK) based on the circRNA-disease association network (Niu *et al.* 2025a, Zhao *et al.* 2026b). Let $A^{cd}\in {\left\{0,1\right\}}^{n_{c}\times n_{d}}$ denote the binary circRNA-disease interaction matrix, where $n_{c}$ and $n_{d}$ denote the numbers of circRNAs and diseases, respectively. Specifically, $A_{ij}^{cd}=1$ if circRNA $c_{i}$ is associated with disease $d_{j}$, and $A_{ij}^{cd}=0$ otherwise. The *i*-th row $A_{i\cdot }^{cd}$ thus represents the interaction profile of circRNA $c_{i}$ across all diseases.

Following the GIPK formulation, we define the functional similarity between two circRNAs $c_{i}$ and $c_{j}$ as:


(1) $$S_{ij}^{\text{circ}}=\text{exp}(-\gamma_{c}||A_{i\cdot }^{cd}-A_{j\cdot }^{cd}|{|}_{2}^{2}),$$

where $\gamma_{c}$ is a bandwidth parameter that controls the decay rate of the kernel. To adapt the kernel scale to the interaction distribution of circRNAs, $\gamma_{c}$ is defined as:


(2) $$\gamma_{c}=\frac{1}{\frac{1}{n_{c}}\sum_{i=1}^{n_{c}}||A_{i\cdot }^{cd}|{|}_{2}^{2}}.$$

#### 3.1.2 Disease ontology-based similarity

We compute disease similarity based on the hierarchical structure of the disease ontology (DO). Each disease term *d* is associated with a set of ancestor terms, denoted as A(d), forming a directed acyclic semantic graph in which contributions attenuate along ontology edges. For each ancestor term a∈A(d), we define its semantic contribution to disease *d* as:


(3) $$S_{d}(a)=\{\begin{array}{cc} 1, & a=d,\\ \underset{a^{\prime }\in \text{child}(a)}{\max}(\lambda_{a,a^{\prime }}S_{d}(a^{\prime })), & a\neq d, \end{array}$$

where $\lambda_{a,a^{\prime }}\in (0,1)$ denotes the attenuation coefficient associated with the ontology edge between ancestor term *a* and its child $a^{\prime }$.

The semantic value of disease *d* is then computed as:


(4) $$\text{SV}(d)=\sum_{a\in A(d)}S_{d}(a),$$

which summarizes the total semantic contribution aggregated from all ancestor terms.

Finally, the semantic similarity between two diseases $d_{i}$ and $d_{j}$ is defined as:


(5) $$S_{\text{DO}}(d_{i},d_{j})=\frac{\sum_{a\in A(d_{i})\cap A(d_{j})}\left(S_{d_{i}}(a)+S_{d_{j}}(a)\right)}{\text{SV}(d_{i})+\text{SV}(d_{j})}.$$

#### 3.1.3 MiRNA sequence embedding

To derive semantic embeddings for miRNA sequences, we employ a pretrained ELECTRA encoder to extract contextual sequence representations (Clark *et al.* 2020). Given an input sequence $s=(s_{1},\ldots ,s_{n})$, a subset of positions M is selected for corruption, where |M|=0.15n following standard masking strategies.

At selected position *r*, the original nucleotide in the miRNA sequence is replaced by a candidate sampled from the generator, which defines a probability distribution over nucleotides at that position:


(6) $$q(u|s,r)=\frac{\;\text{exp}(e{\left(u\right)}^{⊤}g{\left(s\right)}_{r})}{\sum_{v\in \{A,C,G,U\}}\;\text{exp}\;(e{\left(v\right)}^{⊤}g{\left(s\right)}_{r})},$$

where e(u) denotes the embedding of nucleotide *u*, and $g{\left(s\right)}_{r}$ is the generator’s contextual representation.

The discriminator takes the corrupted nucleotide sequence as input and predicts, for position *r*, the probability that the nucleotide has been replaced by the generator:


(7) $$d(s,r)=\sigma \left(w^{⊤}h{\left(s\right)}_{r}\right),$$

where $h{\left(s\right)}_{r}$ denotes the Transformer-based hidden state of the discriminator. The final hidden representation of the discriminator is used as the miRNA sequence embedding.

### 3.2 Diffusion-based prior enhancement

Given the circRNA-disease and miRNA-disease associations, MV-S^2^M constructs a heterogeneous graph G=(V,E), and initializes each node with a prior representation. The node features are denoted as $X^{\left(0\right)}\in R^{|V|\times d}$, where circRNA, disease, and miRNA features are derived from functional similarities, ontology-based semantics, and sequence embeddings, respectively. In the diffusion stage, *A* denotes the adjacency matrix, and $\tilde{A}=D^{-1}A$ represents the row-normalized random-walk transition matrix, where *D* is the degree matrix of *A*. The diffusion process is iteratively performed as:


(8) $$X^{\left(k+1\right)}=(1-\alpha )\tilde{A}X^{\left(k\right)}+\alpha X^{\left(0\right)},$$

where *k* indexes the diffusion iteration, and α∈(0,1) is the restart coefficient.

By recursively expanding the above update rule, the representation after *T* diffusion steps can be expressed as:


(9) $$X^{\left(T\right)}={\left(1-\alpha \right)}^{T}{\tilde{A}}^{T}X^{\left(0\right)}+\alpha \sum_{j=0}^{T-1}{\left(1-\alpha \right)}^{j}{\tilde{A}}^{j}X^{\left(0\right)}.$$

The diffusion-enhanced prior representation $X^{\left(T\right)}$ is subsequently shared across meta-path-guided subgraphs, ensuring that different semantic views inherit consistent prior information.

### 3.3 Multi-view state-space modeling

#### 3.3.1 Semantic subgraph construction

We construct multiple semantic subgraphs based on a set of representative meta-paths P, where p∈P corresponds to a relational pattern including *m*-*d*-*m*, *c*-*d*-*c*, *d*-*m*-*d*, and *d*-*c*-*d*. Let $V_{c}$, $V_{m}$, and $V_{d}$ denote the circRNA, miRNA, and disease node sets, respectively. For each meta-path p∈P, we identify all length-two path instances that follow its prescribed node-type sequence. For example, an *m*-*d*-*m* instance is formed when two distinct miRNAs are associated with the same disease, with mirrored instances treated as identical.

For each meta-path, the semantic view $G^{\left(p\right)}=(V^{\left(p\right)},E^{\left(p\right)})$ is formed by collecting all nodes and original association edges involved in its valid instances. Thus, $V^{\left(p\right)}$ contains the participating endpoint and intermediate nodes, whereas $E^{\left(p\right)}$ retains only the corresponding circRNA-disease or miRNA-disease edges. Duplicate nodes and edges are merged, and no additional direct edges are introduced. The input features for semantic view *p* are obtained by selecting the rows of $X^{\left(T\right)}$ corresponding to $V^{\left(p\right)}$, yielding $X^{\left(p\right)}\in R^{|V^{\left(p\right)}|\times d}$.

#### 3.3.2 State-space modeling

Let $h_{t}^{\left(\ell \right)}\in R^{d}$ denote the representation of the *t*-th node in a semantic sequence at layer ℓ, where $h_{t}^{\left(0\right)}$ is initialized from $X^{\left(p\right)}$. Since the semantic subgraphs do not possess an inherent sequential order, nodes within each view are arranged according to a fixed deterministic ordering to provide a consistent and reproducible input for state-space modeling. The underlying relational structure is encoded through structure-aware diffusion and meta-path-guided subgraph construction. We first compute a feature stream and a gating stream by linear projections:


(10) $$x_{t}^{\left(\ell \right)}=W_{x}h_{t}^{\left(\ell \right)},\;z_{t}^{\left(\ell \right)}=W_{z}h_{t}^{\left(\ell \right)}.$$

where $W_{x}$ and $W_{z}$ are learnable matrices.

We introduce a latent state $s_{t}$ to model the evolution of node representations along the semantic sequence:


(11) $$s_{t}=A_{t}s_{t-1}+B_{t}x_{t}^{\left(\ell \right)},$$

where $A_{t}$ and $B_{t}$ are input-dependent transition parameters. The latent state is then mapped to the output representation through a linear readout:


(12) $$y_{t}=C_{t}s_{t}+Dx_{t}^{\left(\ell \right)},$$

where $C_{t}$ and D are learnable matrices implementing the state-to-output mapping and skip connection, respectively. The output is modulated by the gating signal and projected back into the embedding space:


(13) $${\tilde{y}}_{t}=y_{t}⊙\sigma \left(z_{t}^{\left(\ell \right)}\right),$$

(14) $$h_{t}^{\left(\ell +1\right)}=N\left(h_{t}^{\left(\ell \right)}+{\tilde{y}}_{t}\right),$$

where N(·) denotes layer normalization.

The state-space formulation provides computational advantages for modeling long-range dependencies within semantic views. For a semantic view containing $N_{p}$ nodes, the computation requires $O(N_{p}d^{2})$ time and $O(N_{p}d)$ memory, facilitating scalable representation learning.

#### 3.3.3 Multi-view fusion

Cross-view fusion is important for integrating complementary information from multiple semantic views (Lan *et al.* 2026). By stacking state-space layers, MV-S^2^M obtains subgraph-level representations for semantic view *p*, denoted as $H^{\left(p\right)}\in R^{|V^{\left(p\right)}|\times d}$. For cross-view integration, $H^{\left(p\right)}$ is projected back to the full node space, yielding a view-specific representation matrix $Z^{\left(p\right)}\in R^{N\times d}$.

All view-specific representations are concatenated and fused via a linear transformation:


(15) $$Z=[Z^{\left(1\right)}\|Z^{\left(2\right)}\|Z^{\left(3\right)}\|Z^{\left(4\right)}]W_{f}+b_{f},$$

followed by a residual projection from the initial molecular priors to preserve global prior information and stabilize cross-view fusion:


(16) $$H=N\left(Z+W_{r}X^{\left(0\right)}\right).$$

### 3.4 Downstream task prediction

The learned multi-view node representations are used for zero-shot CMI prediction. For a candidate circRNA-miRNA pair $\left(c_{i},m_{j}\right)$, we retrieve their corresponding node representations from the multi-view fusion matrix *H* and construct a pairwise feature vector by concatenation:


(17) $$z_{ij}=\left[h_{c_{i}}\|h_{m_{j}}\right].$$

The pairwise representation $z_{ij}$ is fed into an MLP classifier to estimate the interaction probability:


(18) $${\hat{y}}_{ij}=\sigma (W_{o}f_{\text{MLP}}(z_{ij})+b_{o}),$$

where $W_{o}$ and $b_{o}$ are learnable parameters, and σ(·) denotes the sigmoid activation function.

The model is optimized using the binary cross-entropy loss:


(19) $$L_{\mathrm{pred}}=-\frac{1}{N}\sum_{\left(i,j\right)}\left[y_{ij}\;\text{log}({\hat{y}}_{ij})+(1-y_{ij})\;\text{log}(1-{\hat{y}}_{ij})\right],$$

where *N* denotes the number of training interaction pairs, $y_{ij}$ and ${\hat{y}}_{ij}$ represent the observed interaction label and predicted probability for the circRNA-miRNA pair $\left(c_{i},m_{j}\right)$, respectively.

## 4 Experiments

### 4.1 Datasets

To assess the ability of MV-S^2^M to infer unseen CMIs, we construct a new dataset, CMI-Zero, in which known CMI edges are excluded from graph representation learning and used only as labels for downstream classifier training and evaluation. Specifically, CMI-Zero is built by integrating circRNA-disease and miRNA-disease associations from publicly available databases, including circAtlas 3.0 (Wu *et al.* 2024), circRNADisease 2.0 (Sun *et al.* 2024), and HMDD 4.0 (Cui *et al.* 2024). During preprocessing, molecular identifiers and disease terms were standardized before cross-database integration, and duplicate associations within and across data sources were removed after standardization. Disease names were further normalized to ensure consistent matching across databases. No additional confidence threshold was applied beyond the inclusion criteria of the original databases. After preprocessing, CMI-Zero contains 442 circRNAs, 310 miRNAs, and 2014 diseases, forming 1812 circRNA-disease and 17 329 miRNA-disease associations. A total of 721 known CMIs are retained as labels for downstream five-fold evaluation, while none of these CMI edges are incorporated into graph construction or representation learning.

We further evaluate generalization performance on CMI-9905 and CMI-9589, two widely used benchmark datasets. CMI-9905, collected from CircBank and circR2Cancer, contains 2346 circRNAs, 962 miRNAs, and 9905 interactions (Liu *et al.* 2019, Lan *et al.* 2020). CMI-9589, derived from CircBank, includes 2115 circRNAs, 821 miRNAs, and 9589 interactions.

### 4.2 Baselines

We compare MV-S^2^M with three categories of baselines: graph representation learning methods, including SDNE (Wang *et al.* 2016), MUSAE (Rozemberczki *et al.* 2021), and GDC (Gasteiger *et al.* 2019); graph neural network methods, including HGT (Hu *et al.* 2020), MetaGC (Jo *et al.* 2023), and ADA-GAD (He *et al.* 2024); and multi-source feature fusion models for CMI prediction, including BioKG-CMI (Wei *et al.* 2024), EGATCMI (Wei *et al.* 2025), MFERL (Peng *et al.* 2025), and HMA-GCA (Hu *et al.* 2026).

Graph representation learning methods exploit graph structures through embedding or diffusion techniques. SDNE preserves first- and second-order graph proximities using a deep autoencoder; MUSAE learns multi-scale representations by aggregating neighborhood attributes through random walks; and GDC enhances node representations through graph diffusion. Graph neural network methods learn node representations through message passing or attention mechanisms. HGT employs type-dependent attention to model heterogeneous graph structures; MetaGC adaptively reweights node pairs through meta-learning to reduce structural noise; ADA-GAD learns representations from anomaly-reduced graphs using pretrained graph autoencoders; and HMA-GCA integrates sequence and topological features through hybrid manifold augmentation and gated cross-attention for CMI prediction. Multi-source feature fusion methods integrate complementary biological information for CMI prediction. BioKG-CMI integrates molecular sequences and relational information through a biological knowledge graph; EGATCMI combines Transformer-based sequence features with graph attention-based topological representations; and MFERL integrates homogeneous and heterogeneous molecular features through multi-scale representation and contrastive learning.

### 4.3 Negative sampling and evaluation

Negative samples are constructed using a biologically constrained strategy based on subcellular localization predicted by lncLocator and miRNALoc (Cao *et al.* 2018, Meher *et al.* 2020). Candidate pairs sharing the same predicted cellular compartment are excluded, and negative samples are randomly selected from the remaining candidates to match the number of positive samples.

In this study, we use Accuracy (Acc.), F1-score (F1), the Area Under the Receiver Operating Characteristic Curve (AUC), and the Area Under the Precision-Recall Curve (AUPR) as evaluation metrics under five-fold cross-validation Zhao *et al.* (2026a). Different representation learning settings are adopted for the zero-shot and conventional scenarios. In CMI-Zero, circRNA-miRNA links are excluded from graph construction, and representations are learned solely from circRNA-disease and miRNA-disease associations. For CMI-9905 and CMI-9589, the conventional setting uses the available CMI network for representation learning, following the standard evaluation protocol of existing CMI prediction studies.

### 4.4 Implementation details

For CMI-Zero, the diffusion-based prior enhancement in MV-S^2^M uses diffusion hops T=15 and a restart coefficient α=0.2. The node embedding dimension is set to d=64, and three state-space layers are stacked for each semantic subgraph. Model parameters are optimized using the Adam optimizer with a learning rate of 0.001 and a weight decay of $1\times 10^{-5}$. The model is trained for 200 epochs, with performance monitored on a validation split. For CMI-9905 and CMI-9589, the same tuning strategy is applied independently to determine dataset-specific settings. To reduce variability introduced by stochastic optimization, all experiments are conducted with fixed random seeds where applicable. Experiments are conducted on a workstation with an NVIDIA GeForce RTX 5070 GPU.

### 4.5 Main results

We evaluate MV-S^2^M against baselines on three benchmark datasets. As shown in Table 1, MV-S^2^M outperforms competing methods across all evaluation metrics. The performance gains are most evident on CMI-Zero, where MV-S^2^M achieves an AUC of 0.9643, with an absolute improvement of 2.14 percentage points over the best competing method. Beyond the zero-shot setting, MV-S^2^M also demonstrates strong performance on benchmark datasets. On CMI-9905 and CMI-9589, the proposed model achieves AUC scores of 0.9837 and 0.9863, respectively. Compared with the best baselines, MV-S^2^M obtains AUC improvements of 2.07 and 1.50 percentage points on these two datasets, while consistently achieving the best Acc., F1, AUC, and AUPR scores among all compared methods. These results indicate that MV-S^2^M learns discriminative and generalizable representations for CMI prediction. By integrating diffusion-enhanced prior knowledge with state-space modeling, the framework effectively exploits complementary biological contexts and long-range relational dependencies, leading to superior performance across diverse benchmark settings.

**Table 1:** Performance comparison of MV-S^2^M with baseline methods on three datasets under five-fold cross-validation.

Method	CMI-Zero				CMI-9905				CMI-9589			
	Acc.	F1	AUC	AUPR	Acc.	F1	AUC	AUPR	Acc.	F1	AUC	AUPR
SDNE	0.8245	0.8219	0.9078	0.9165	0.8591	0.8343	0.8927	0.9018	0.8342	0.8297	0.9153	0.9238
	(±3.24)	(±2.89)	(±2.11)	(±1.36)	(±6.32)	(±9.25)	(±7.12)	(±7.37)	(±0.88)	(±0.94)	(±0.48)	(±0.54)
MUSAE	0.8502	0.8459	0.9039	0.9154	0.8601	0.8370	0.9072	0.8923	0.8962	0.8985	0.9529	0.9629
	(±3.22)	(±3.42)	(±2.50)	(±3.23)	(±2.24)	(±3.12)	(±2.35)	(±2.52)	(±4.47)	(±3.77)	(±1.80)	(±1.61)
GDC	0.8287	0.8228	0.8956	0.9206	0.8297	0.8430	0.9194	0.9031	0.8370	0.8502	0.9266	0.9052
	(±2.12)	(±1.85)	(±1.57)	(±1.13)	(±3.38)	(±2.18)	(±2.47)	(±4.24)	(±3.45)	(±1.81)	(±2.61)	(±4.66)
HGT	0.8322	0.8323	0.9133	0.9341	0.8646	0.8710	0.9347	0.9166	0.8543	0.8599	0.9306	0.9187
	(±6.39)	(±3.70)	(±0.92)	(±0.82)	(±2.25)	(±1.42)	(±2.51)	(±4.24)	(±1.07)	(±1.40)	(±1.10)	(±1.62)
MetaGC	0.8398	0.8275	0.9068	0.9311	0.8342	0.8292	0.9198	0.9274	0.8966	0.8955	0.9641	0.9687
	(±2.13)	(±2.82)	(±1.20)	(±0.87)	(±1.15)	(±1.81)	(±0.66)	(±0.67)	(±0.27)	(±0.31)	(±0.16)	(±0.15)
ADA-GAD	0.8377	0.8360	0.9092	0.9253	0.8519	0.8522	0.9356	0.9316	0.8603	0.8515	0.9473	0.9495
	(±0.67)	(±1.29)	(±0.93)	(±1.86)	(±4.82)	(±4.81)	(±2.69)	(±3.54)	(±2.03)	(±2.95)	(±0.44)	(±0.56)
BioKG-CMI	0.8301	0.8159	0.8935	0.9212	0.8219	0.7923	0.9063	0.9278	0.8561	0.8358	0.9463	0.9581
	(±2.25)	(±2.35)	(±1.69)	(±1.28)	(±1.70)	(±2.66)	(±1.40)	(±0.93)	(±0.96)	(±1.19)	(±0.35)	(±0.25)
EGATCMI	0.8634	0.8592	0.9249	0.9438	0.8277	0.8397	0.9106	0.8967	0.8778	0.8798	0.9470	0.9426
	(±1.37)	(±1.13)	(±1.16)	(±0.65)	(±0.35)	(±0.24)	(±0.22)	(±0.53)	(±0.27)	(±0.28)	(±0.26)	(±0.34)
MFERL	0.8731	0.8817	0.9144	0.9156	0.9084	0.9074	0.9596	0.9610	0.9170	0.9177	0.9669	0.9629
	( ±7.53)	( ±6.79)	(±4.53)	(±4.67)	(±0.54)	(±0.65)	(±0.28)	( ±0.41)	(±0.55)	(±0.63)	(±0.40)	(±0.50)
HMA-GCA	0.8655	0.8444	0.9429	0.9616	0.9088	0.9098	0.9630	0.9609	0.9237	0.9256	0.9713	0.9695
	(±2.85)	(±4.03)	( ±2.25)	( ±1.54)	( ±0.54)	( ±0.50)	( ±0.21)	(±0.25)	( ±0.19)	( ±0.20)	( ±0.13)	( ±0.17)
MV-S^2^M	**0.9411**	**0.9391**	**0.9643**	**0.9760**	**0.9343**	**0.9342**	**0.9837**	**0.9851**	**0.9435**	**0.9429**	**0.9863**	**0.9854**
	**(** ± **0.43)**	**(** ± **0.58)**	**(** ± **0.59)**	**(** ± **0.37)**	**(** ± **0.72)**	**(** ± **0.83)**	**(** ± **0.31)**	**(** ± **0.27)**	**(** ± **1.35)**	**(** ± **1.28)**	**(** ± **0.42)**	**(** ± **0.45)**
*Improv.(%)*	6.80	5.74	2.14	1.44	2.55	2.44	2.07	2.41	1.98	1.73	1.50	1.59

Results are reported as mean and standard deviation over five folds (std in %). The best results are highlighted in bold, while the second-best results are underlined.

### 4.6 Ablation study

We conduct an ablation study to examine the contribution of individual components in MV-S^2^M. Three variants are considered: removing state-space modeling (w/o SSM), removing diffusion-based prior enhancement (w/o Diff), and removing the residual pathway (w/o Res). Their performance is compared with MV-S^2^M on CMI-Zero, CMI-9905, and CMI-9589, as illustrated in Fig. 2.

**Figure 2:**
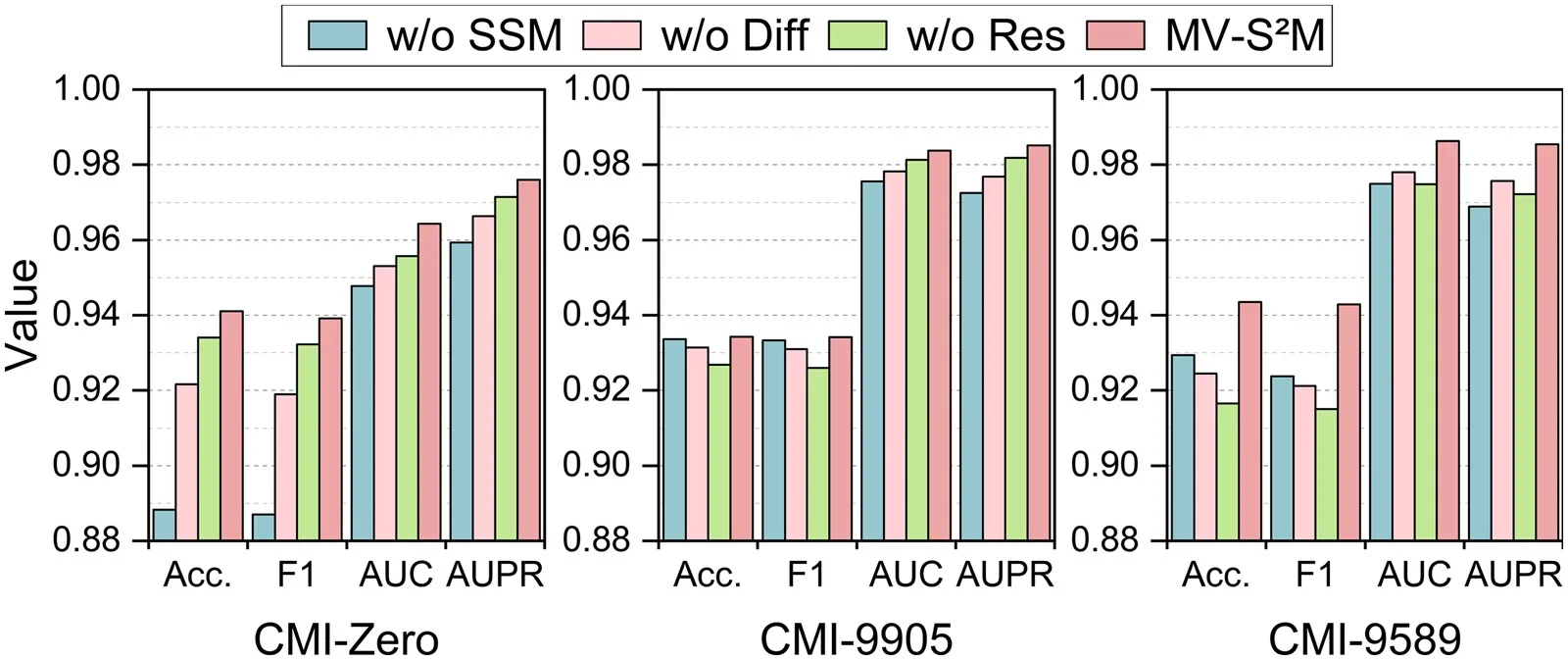
Ablation study of MV-S^2^M on three benchmark datasets.

Across all datasets, the ablation variants exhibit performance degradation compared with the full model, with the most pronounced drops observed on CMI-Zero. In particular, w/o SSM causes substantial declines in Acc. and F1, highlighting that the long-range relational dependencies captured by state-space modeling play a critical role in achieving accurate CMI prediction. Likewise, w/o Diff yields noticeable performance losses, indicating the effectiveness of propagating molecular priors through structure-aware diffusion. In contrast, w/o Res leads to performance degradation, with evident drops on CMI-9905 and CMI-9589 for several metrics, suggesting the importance of residual connections in stabilizing feature integration across datasets. These results demonstrate that each component makes a distinct contribution to the model, and their combination is essential for ensuring competitive performance.

### 4.7 Hyperparameter sensitivity analysis

We analyse the sensitivity of MV-S^2^M to key hyperparameters. Specifically, the investigated hyperparameters are grouped into two categories: architectural hyperparameters, including the number of state-space layers and diffusion hops, and training hyperparameters, including the learning rate and the number of training epochs.

- *Architectural hyperparameters.* As shown in Fig. 3a, performance improves with deeper state-space modeling and peaks at three state-space layers, after which over-parameterization leads to degradation. Increasing diffusion hops enhances performance up to T=15, reflecting effective propagation of molecular priors across the network structure, while additional hops lead to redundant propagation and performance degradation.
- *Training hyperparameters.*  Figure 3b shows that a learning rate of 0.001 leads to the most consistent performance, whereas smaller or larger values achieve relatively lower performance. As training epochs increase, performance steadily improves and reaches its peak around 200 epochs. Further increasing the number of epochs leads to performance degradation, indicating that excessive training may negatively affect model generalization.

**Figure 3:**
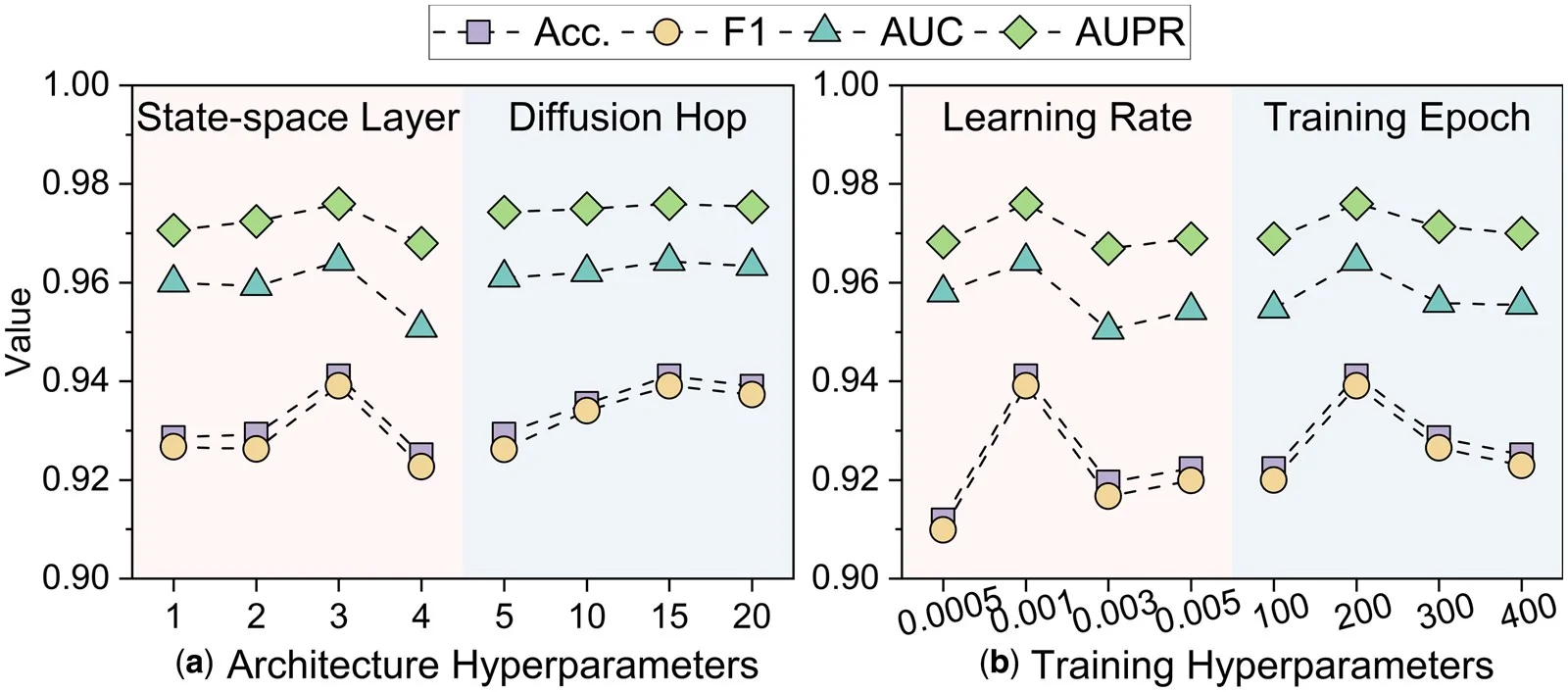
Sensitivity analysis of MV-S^2^M on CMI-Zero under different hyperparameter settings. (a) Impact of architectural hyperparameters. (b) Impact of training hyperparameters.

Overall, MV-S^2^M maintains stable performance across a reasonable range of hyperparameter settings, indicating the reliability of the proposed framework.

### 4.8 Entity-level cold-start evaluation

The relation-level zero-shot setting evaluates the ability of MV-S^2^M to infer unobserved CMIs without incorporating known links into graph representation learning. To further examine whether the learned representations can generalize to previously unseen molecular entities, we conduct additional entity-level cold-start experiments. Specifically, we consider three increasingly challenging settings. In the *Unseen circRNA* setting, 10% of circRNAs are randomly selected as unseen entities, and all interaction pairs involving these circRNAs are excluded from training and used exclusively for testing. The *Unseen miRNA* setting is constructed analogously by holding out 10% of miRNAs. Finally, the *Double unseen* setting simultaneously holds out 10% of circRNAs and 10% of miRNAs, where a test pair is retained only if both endpoints are unseen during training. Samples containing only one unseen endpoint are discarded to avoid information leakage.

Table 2 reports the results under the entity-level cold-start settings. MV-S^2^M achieves strong performance in the *Unseen circRNA* setting, with an AUC of 0.9536 and an AUPR of 0.9682. In contrast, performance decreases substantially in the *Unseen miRNA* setting and shows considerably larger variation across different splits, indicating that generalization to unseen miRNAs is more challenging. This weaker and less stable performance may be related to the denser miRNA–disease association structure, which makes representation learning more sensitive when miRNAs are held out. The *Double unseen* setting is more restrictive because neither endpoint of a test interaction is observed during training, and it also exhibits relatively high variability. Overall, these results show that the cold-start generalization of MV-S^2^M differs between circRNAs and miRNAs, with unseen miRNAs and double-unseen interactions remaining more challenging.

**Table 2:** Performance of MV-S^2^M under entity-level cold-start settings.

Setting	Acc.	F1	AUC	AUPR
Unseen circRNA	0.9203	0.9155	0.9536	0.9682
	(±1.99)	(±2.63)	(±2.06)	(±1.44)
Unseen miRNA	0.6320	0.7337	0.5403	0.7212
	(±13.90)	(±8.96)	(±32.55)	(±16.29)
Double unseen	0.6023	0.6661	0.7462	0.7940
	(±13.81)	(±10.99)	(±30.59)	(±28.17)
Standard split	0.9411	0.9391	0.9643	0.9760
	(±0.43)	(±0.58)	(±0.59)	(±0.37)

Results are reported as mean and standard deviation over five random splits (std in %).

### 4.9 Comparison of negative sampling strategies

Since unknown circRNA-miRNA pairs may contain undiscovered positive interactions, different negative sampling strategies may lead to different prediction results. Therefore, we compare the predicted scores assigned to experimentally verified positive interactions under random negative sampling and biologically constrained negative sampling.

Table 3 summarizes the results. The reported Random and Bio-Constrained values represent the average prediction scores of positive interactions under the two negative sampling strategies. Across all three datasets, models with biologically constrained negative sampling produce higher prediction scores than models with random sampling. For example, the average prediction score on CMI-Zero increases from 0.5533 to 0.8911 after introducing subcellular localization constraints. Similar improvements are observed on CMI-9589 and CMI-9905. Larger score discrepancies between the two strategies tend to coincide with lower Spearman correlations, indicating that negative sampling choices affect prediction rankings. Moreover, Wilcoxon signed-rank tests show statistically significant differences (*P* < .001) across all datasets, indicating that the choice of negative samples has a considerable impact on model predictions.

**Table 3:** Comparison of model predictions under different negative sampling strategies.

Dataset	Random	Bio-constrained	Spearman	Wilcoxon *P*
CMI-Zero	0.5533	0.8911	0.1689	<.001
CMI-9589	0.9096	0.9734	0.3790	<.001
CMI-9905	0.8731	0.9690	0.4067	<.001

### 4.10 Case study on HCC-related CMI prediction

To further examine the biological relevance of the predictions generated by MV-S^2^M, we performed an additional case study on HCC. Specifically, all candidate circRNA-miRNA pairs associated with HCC were ranked according to their prediction scores, and the top-ranked interactions were manually examined through PubMed.

Table 4 lists the top 10 predicted HCC-related CMIs. Among them, eight interactions have been supported by existing literature, while the remaining two pairs have not yet been reported. These two interactions should therefore be regarded as candidate HCC-related CMIs that require further experimental verification. Overall, the literature-supported predictions provide additional evidence for the biological relevance of the model outputs, while the unsupported pairs may serve as candidates for future investigation.

**Table 4:** Top 10 predicted HCC-related CMIs.

Rank	CircRNA	MiRNA	Evidence (PMID)
1	circ_0005224	miR-29a	34,795,497
2	circ_0005397	miR-382	30,444,423
3	circ_0002891	miR-1228	N/A
4	circ_0000284	miR-107	33,015,172
5	circ_0072088	miR-1270	33,928,018
6	circ_0001946	miR-7	28,892,615
7	circ_0001821	miR-145	33,728,345
8	circ_0067934	miR-335	33,716,239
9	circ_0003222	miR-200b	N/A
10	circ_005239	miR-1228	34,668,628

### 4.11 Contribution of disease-mediated semantic subgraphs

We conduct case studies to investigate the contribution of different disease-mediated semantic subgraphs to zero-shot CMI prediction, using hepatocellular carcinoma (HCC) as a representative example. Figure 4a compares MV-S^2^M with variants obtained by individually removing different semantic subgraphs. Performance degradation is observed across these variants, particularly for the *d*-*c*-*d* and *m*-*d*-*m* views. To further distinguish their effects, we select five HCC-related circRNA-miRNA pairs with high prediction confidence for detailed analysis. The corresponding prediction scores exhibit substantially larger variations when the *m*-*d*-*m* subgraph is removed, whereas removing *d*-*c*-*d* subgraphs results in relatively minor changes.

**Figure 4:**
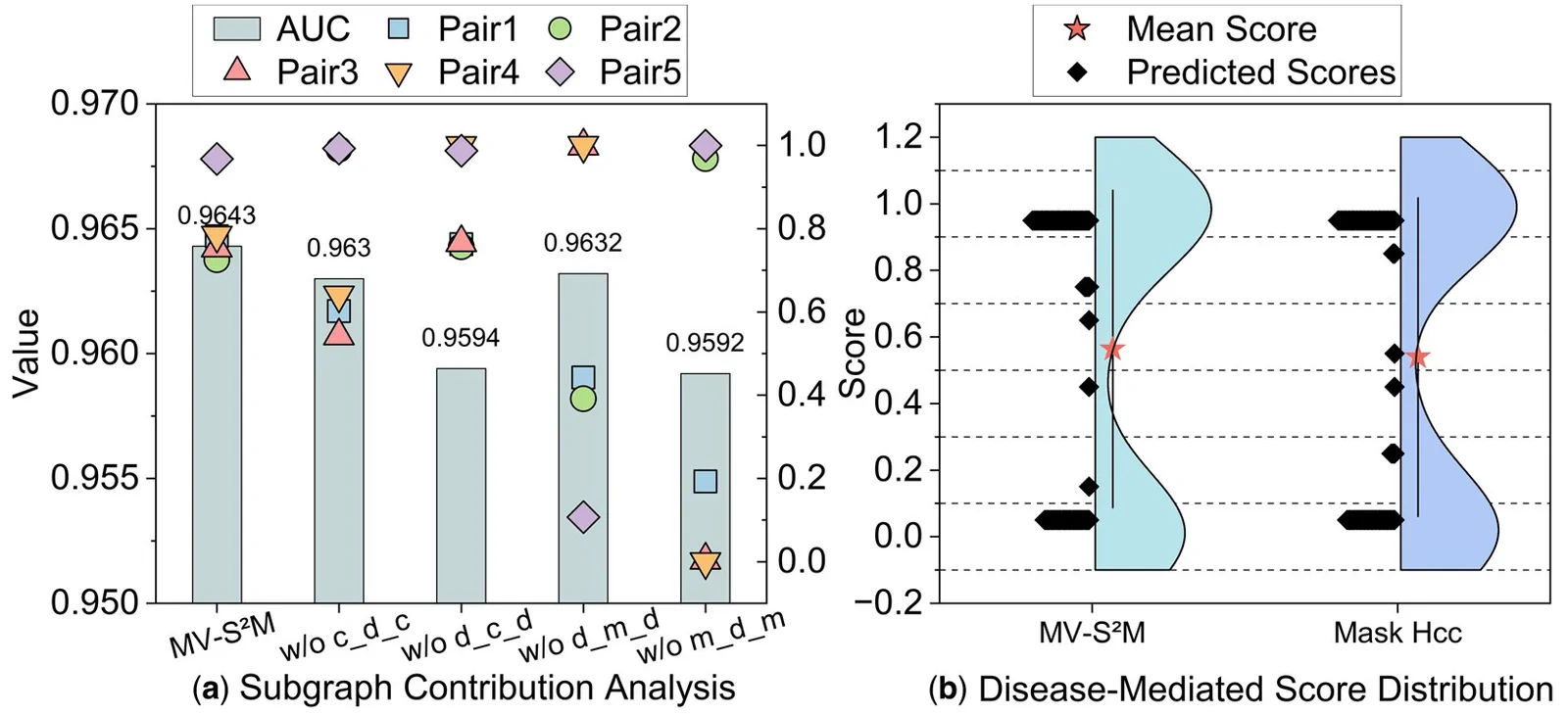
Case studies on disease-mediated semantic subgraphs for zero-shot CMI prediction. (a) MV-S^2^M vs. subgraph-removed variants, with AUC and prediction score changes for representative HCC-related circRNA-miRNA pairs. (b) Prediction score distributions before and after masking HCC-related information.

To better understand how disease information affects prediction behavior, we mask HCC-related edges within the *m*-*d*-*m* subgraph and analyse the resulting score distributions of HCC-associated positive samples. As shown in Fig. 4b, masking HCC-related edges shifts the prediction scores toward lower values, while the overall distribution pattern remains broadly similar. Taken together, this case study demonstrates that meta-path-guided semantic subgraphs, especially the *m*-*d*-*m* view, play a crucial role in CMI prediction. Disease signals enhance the robustness of molecular representations, enabling MV-S^2^M to generalize effectively to previously unseen circRNA-miRNA pairs.

## 5 Conclusion

In this paper, we propose MV-S^2^M, a state-space modeling framework for zero-shot CMI prediction. MV-S^2^M incorporates circRNA-disease and miRNA-disease associations to provide disease-level regulatory context for representation learning. It enriches molecular priors through structure-aware diffusion and encodes disease-mediated semantic views with stacked state-space layers to capture long-range relational dependencies. Extensive experiments on three benchmark datasets demonstrate that MV-S^2^M consistently outperforms baseline methods, while ablation and sensitivity analyses validate the effectiveness and stability of the proposed framework. Cold-start evaluations additionally confirm the generalization ability under unseen entity settings. Case studies further illustrate the contribution of disease-mediated relational information to model predictions.

In future work, we plan to extend the model with richer biological evidence and explore more transferable representation learning strategies to improve scalability and interpretability. Beyond CMI prediction, the general framework of MV-S^2^M may also be extended to other non-coding RNA interaction and association prediction tasks. In such settings, task-specific molecular priors and meta-paths can be constructed from the available biological relations, while the multi-view state-space modeling strategy can be retained to characterize complementary and long-range relational dependencies.

## Data Availability

The code and data are available at https://github.com/591286260/MV and https://doi.org/10.5281/zenodo.22858403.

## References

[btag797-B1] Awata D , Radhakrishnan V, Shaath H et al Circular rna profiling identifies circ_0001522, circ_0001278, and circ_0001801 as predictors of unfavorable prognosis and drivers of triple-negative breast cancer hallmarks. Cell Death Discov 2025;11:316. 10.1038/s41420-025-02576-9 PMC12241340 40634303

[btag797-B2] Cao C , Wang C, Dai Q et al Crbpsa: circrna-rbp interaction sites identification using sequence structural attention model. BMC Biol 2024;22:260. 10.1186/s12915-024-02055-0 PMC11566611 39543602

[btag797-B3] Cao C , Li M, Wang C et al Dgclcmi: a deep graph collaboration learning method to predict circrna-mirna interactions. BMC Biol 2025;23:104. 10.1186/s12915-025-02197-9 PMC12016396 40264118

[btag797-B4] Cao Z , Pan X, Yang Y et al The lnclocator: a subcellular localization predictor for long non-coding rnas based on a stacked ensemble classifier. Bioinformatics 2018;34:2185–94. 10.1093/bioinformatics/bty085 29462250

[btag797-B5] Char S , Corley N, Alamdari S et al Protnote: a multimodal method for protein–function annotation. Bioinformatics 2025;41:btaf170. 10.1093/bioinformatics/btaf170 PMC12054973 40233101

[btag797-B6] Chen L , Chen Y, Zhou B. Hclamcmi: prediction of circrna–mirna interactions based on hypergraph contrastive learning and an attention mechanism. J Chem Inf Model 2025;65:12099–115. 10.1021/acs.jcim.5c01968 41108288

[btag797-B7] Chen Z , Ji M, Qian J et al Probid-net: a deep learning model for protein–protein binding interface design. Chem Sci 2024;15:19977–90. 10.1039/d4sc02233e PMC11575592 39568891

[btag797-B8] Cheng L , Zhu P, Guo Y et al Sourcedetmamba: a graph-aware state space model for source detection in sequential hypergraphs. In Kwok J (ed.), *Proceedings of the Thirty-Fourth International Joint Conference on Artificial Intelligence, IJCAI-25*, pp. 2749–57. International Joint Conferences on Artificial Intelligence Organization, 2025. 10.24963/ijcai.2025/306

[btag797-B9] Clark K , Luong M-T, Le QV et al Electra: pre-training text encoders as discriminators rather than generators. In: *International Conference on Learning Representations*, pp 3295–312. 2020.

[btag797-B10] Cui C , Zhong B, Fan R et al Hmdd v4. 0: a database for experimentally supported human microrna-disease associations. Nucleic Acids Res 2024;52:D1327–32. 10.1093/nar/gkad717 PMC10767894 37650649

[btag797-B11] Fang Z , Lei X. Prediction of mirna-circrna associations based on k-nn multi-label with random walk restart on a heterogeneous network. Big Data Min Anal 2019;2:261–72. 10.26599/BDMA.2019.9020010

[btag797-B12] Gasteiger J , Weiß Enberger S, Günnemann S. Diffusion improves graph learning. In: Wallach H, Larochelle H, Beygelzimer A, Alché-Buc F, Fox E, Garnett R (eds.), Advances in Neural Information Processing Systems. Vol. 32. Red Hook, NY, USA: Curran Associates, Inc., 2019.

[btag797-B13] Hashemi M , Khosroshahi EM, Daneii P et al Emerging roles of circrna-mirna networks in cancer development and therapeutic response. Noncoding RNA Res 2025;10:98–115. 10.1016/j.ncrna.2024.09.006 PMC11440256 39351450

[btag797-B14] He J , Xu Q, Jiang Y et al Ada-gad: Anomaly-denoised autoencoders for graph anomaly detection. In: *Proceedings of the AAAI Conference on Artificial Intelligence*, vol. 38, pp. 8481–9, 2024.

[btag797-B15] Hu Y , Wang Y, Bai Y et al Hma-gca: hybrid manifold augmentation and gated cross-attention for circrna-mirna interaction prediction. Bioinformatics 2026;42:btag536. 10.1093/bioinformatics/btag536 PMC13440667 42474246

[btag797-B16] Hu Z , Dong Y, Wang K et al Heterogeneous graph transformer. In: *Proceedings of The Web Conference 2020*, WWW ’20, pp. 2704–2710. New York, NY, USA: Association for Computing Machinery. 2020. 10.1145/3366423.3380027

[btag797-B17] Jo H , Bu F, Shin K. Robust graph clustering via meta weighting for noisy graphs. In: *Proceedings of the 32nd ACM International Conference on Information and Knowledge Management*, pp. 1035–44. 2023.

[btag797-B18] Lan W , Zhu M, Chen Q et al Circr2cancer: a manually curated database of associations between circrnas and cancers. Database 2020;2020:baaa085. 10.1093/database/baaa085 PMC7661096 33181824

[btag797-B19] Lan W , Guo Y, Chen Q et al Cafe: cross-view adaptive fusion and cluster center enhancement for robust multi-view clustering. IEEE Trans Pattern Anal Mach Intell 2026;48:13653–68. 10.1109/TPAMI.2026.3708284 42371887

[btag797-B20] Li J , Wu R, Jin X et al State space models on temporal graphs: a first-principles study. In Globerson A, Mackey L, Belgrave D, Fan A, Paquet U, Tomczak J, Zhang C (eds.), Advances in Neural Information Processing Systems. Vol. 37. Red Hook, NY, USA: Curran Associates, Inc., 2024, 127030–58. 10.52202/079017-4034

[btag797-B21] Liu M , Wang Q, Shen J et al Circbank: a comprehensive database for circrna with standard nomenclature. RNA Biol 2019;16:899–905. 10.1080/15476286.2019.1600395 PMC6546381 31023147

[btag797-B22] Ma Z , Kuang Z, Deng L. Ngcicm: a novel deep learning-based method for predicting circrna-mirna interactions. IEEE/ACM Trans Comput Biol Bioinform 2023;20:3080–92. 10.1109/TCBB.2023.3248787 37027645

[btag797-B23] Meher PK , Satpathy S, Rao AR. Mirnaloc: predicting mirna subcellular localizations based on principal component scores of physico-chemical properties and pseudo compositions of di-nucleotides. Sci Rep 2020;10:14557. 10.1038/s41598-020-71381-4 PMC7471944 32884018

[btag797-B24] Niu M , Chen Y, Wang C et al Computational approaches for circrna-disease association prediction: a review. Front Comput Sci 2025a;19:194904. 10.1007/s11704-024-40060-2

[btag797-B25] Niu M , Wang C, Chen Y et al Interpretable multi-instance heterogeneous graph network learning modelling circrna-drug sensitivity association prediction. BMC Biol 2025b;23:131. 10.1186/s12915-025-02223-w PMC12079948 40369616

[btag797-B26] Peng L , Wang W, Yang Z et al Leveraging explainable multi-scale features for fine-grained circrna-mirna interaction prediction. BMC Biol 2025;23:121. 10.1186/s12915-025-02227-6 PMC12065178 40346538

[btag797-B27] Ren L , Liu Y, Lu Y et al Samba: simple hybrid state space models for efficient unlimited context language modeling. In: *The Thirteenth International Conference on Learning Representations*. 2025.

[btag797-B28] Rozemberczki B , Allen C, Sarkar R. Multi-scale attributed node embedding. J Complex Netw 2021;9:cnab014. 10.1093/comnet/cnab014

[btag797-B29] Sun Z-Y , Yang C-L, Huang L-J et al Circrnadisease v2. 0: an updated resource for high-quality experimentally supported circrna-disease associations. Nucleic Acids Res 2024;52:D1193–200. 10.1093/nar/gkad949 PMC10767896 37897359

[btag797-B30] Tan L , Zhao W, Wang H et al Dysfunctional circular rna network in major depressive disorder: dissecting the cell identity and potential clinical applications. Mol Psychiatry 2026;31:498–513. 10.1038/s41380-025-03167-x 40847006

[btag797-B31] Wang D , Cui P, Zhu W. Structural deep network embedding. In: *Proceedings of the 22nd ACM SIGKDD International Conference on Knowledge Discovery and Data Mining*, pp. 1225–34. 2016. 10.1145/2783258.2783288 PMC4688012 26705505

[btag797-B32] Wang X-F , Yu C-Q, You Z-H et al A feature extraction method based on noise reduction for circrna-mirna interaction prediction combining multi-structure features in the association networks. Brief Bioinform 2023a;24:bbad111. 10.1093/bib/bbad111 36971393

[btag797-B33] Wang X-F , Yu C-Q, You Z-H et al Berolecmi: a novel prediction method to infer circrna-mirna interaction from the role definition of molecular attributes and biological networks. BMC Bioinformatics 2024a;25:264. 10.1186/s12859-024-05891-7 PMC11316391 39127625

[btag797-B34] Wang Y , Xia Y, Yan J et al Zerobind: a protein-specific zero-shot predictor with subgraph matching for drug-target interactions. Nat Commun 2023b;14:7861. 10.1038/s41467-023-43597-1 PMC10687269 38030641

[btag797-B35] Wang Z , Xiong Z, Huang F et al Zeroddi: a zero-shot drug-drug interaction event prediction method with semantic enhanced learning and dual-modal uniform alignment. IJCAI ’24. 2024b. 10.24963/ijcai.2024/671

[btag797-B36] Wei M , Wang L, Li Y et al Biokg-cmi: a multi-source feature fusion model based on biological knowledge graph for predicting circrna-mirna interactions. Sci China Inf Sci 2024;67:189104. 10.1007/s11432-024-4098-3

[btag797-B37] Wei M , Wang L, Su X et al Multi-hop graph structural modeling for cancer-related circrna-mirna interaction prediction. Pattern Recognit 2026;170:112078. 10.1016/j.patcog.2025.112078

[btag797-B38] Wei M-M , Wang L, Zhao B-W et al Integrating transformer and graph attention network for circrna-mirna interaction prediction. IEEE J Biomed Health Inform 2025;29:6105–13. 10.1109/JBHI.2025.3561197 40232907

[btag797-B39] Wu W , Zhao F, Zhang J. Circatlas 3.0: a gateway to 3 million curated vertebrate circular rnas based on a standardized nomenclature scheme. Nucleic Acids Res 2024;52:D52–60. 10.1093/nar/gkad770 PMC10767913 37739414

[btag797-B40] Xu H , Woicik A, Poon H et al Multilingual translation for zero-shot biomedical classification using biotranslator. Nat Commun 2023;14:738. 10.1038/s41467-023-36476-2 PMC9911740 36759510

[btag797-B41] Yin W , Wang S, Zhang Y et al Autonomously adjusting multi-relational hypergraphs structure for predicting circrna-mirna associations. IEEE J Biomed Health Inform 2026;30:8244–53. 10.1109/JBHI.2025.3531427 40031229

[btag797-B42] Zhang X , Zou Q, Niu M et al Predicting circrna–disease associations with shared units and multi-channel attention mechanisms. Bioinformatics 2025;41:btaf088. 10.1093/bioinformatics/btaf088 PMC11919450 40045181

[btag797-B43] Zhao B , Li D, Yang Y et al Drhin: an integrated and interactive web server for drug repositioning. J Chem Inf Model 2026a;66:5008–17. 10.1021/acs.jcim.6c00311 42001407

[btag797-B44] Zhao B-W , Zhao H-F, Huang Y-A et al A novel graph transformer framework for predicting drug-disease associations with structural awareness. IEEE Trans Comput Biol Bioinform 2026b;23:2114–25. 10.1109/TCBBIO.2026.3721658 42560912

[btag797-B45] Zhou J , Niu R, Ji B et al A knowledge graph enhanced pre-trained large language model for predicting microrna-circrna interactions. Big Data Min Anal 2025;8:1405–17. 10.26599/BDMA.2025.9020014

[btag797-B46] Zhu X , Wang L, Tang R et al Spatial-spectral fusion enables drug repositioning by capturing indirect and long-range associations in biological networks. Bioinformatics 2026;42:btag553. 10.1093/bioinformatics/btag553 PMC13461371 42573507

